# Network Pharmacology and Molecular Docking-Based Analysis on Bioactive Anticoronary Heart Disease Compounds in *Trichosanthes kirilowii* Maxim and *Bulbus allii* Macrostemi

**DOI:** 10.1155/2021/6704798

**Published:** 2021-11-16

**Authors:** Yi-Ding Yu, Wang-Jun Hou, Juan Zhang, Yi-Tao Xue, Yan Li

**Affiliations:** ^1^Shandong University of Traditional Chinese Medicine, Jinan 250014, China; ^2^Affiliated Hospital of Shandong University of Traditional Chinese Medicine, Jinan 250014, China

## Abstract

*Trichosanthes kirilowii* Maxim. and *Bulbus allii* Macrostemi are the components of Gualou Xiebai decoction (GLXB), a commonly used herbal combination for the treatment of coronary heart disease (CHD) in traditional Chinese medicine. Although GLXB is associated with a good clinical effect, its active compounds and mechanism of action remain unclear, which limits its clinical application and the development of novel drugs. In this study, we explored key compounds, targets, and mechanisms of action for GLXB in the treatment of CHD using the network pharmacology approach. We identified 18 compounds and 21 action targets via database screening. Enrichment analysis indicated that the effects of GLXB in patients with CHD are primarily associated with the regulation of signalling pathways for tumour necrosis factor, nuclear factor-kappa B, hypoxia-inducible factor-1, arachidonic acid metabolism, and insulin resistance. GLXB thus exerts anti-inflammatory, antihypoxic, and antiagglutinating effects; regulates lipid metabolism; and combats insulin resistance in CHD via these pathways, respectively. After reverse targeting, we observed that the main active compounds of GLXB in the treatment of CHD were quercetin, naringenin, *β*-sitosterol, ethyl linolenate, ethyl linoleate, and prostaglandin B1. To explore the potential of these compounds in the treatment of CHD, we verified the affinity of the compounds and targets via molecular docking analysis. Our study provides a bridge for the transformation of natural herbs and molecular compounds into novel drug therapies for CHD.

## 1. Introduction

Coronary heart disease (CHD) is characterised by atherosclerosis in the coronary arteries, leading to blockage of the vascular lumen and subsequent myocardial ischaemia, hypoxia, and necrosis. In the 2016 update on Heart Disease and Stroke Statistics, the American Heart Association reported that 15.5 million individuals aged ≥20 years in the USA have CHD [1]. Thus, in addition to its severe effects on patient quality of life, CHD is associated with a significant societal and economic burden. Moreover, long-term use of aspirin, statins, and other drugs can have adverse effects on health [2, 3], making it necessary to identify alternative treatment strategies with a reduced risk of side effects.

In recent years, the field of drug research and development has placed an emphasis on identifying natural herbal medicines that can improve patient outcomes without the adverse effects of standard pharmacological agents. Gualou Xiebai decoction (GLXB) has long been used as a treatment for symptoms of chest pain in traditional Chinese medicine (TCM). However, due to the complexity of TCM compounds, multiple target sites, and diverse pathways of action, the previous research model—in which studies aim to identify unique compounds that exhibit regulatory activity at exclusive sites—cannot be used to adequately explain the overall role of TCM.

**Figure 1:**
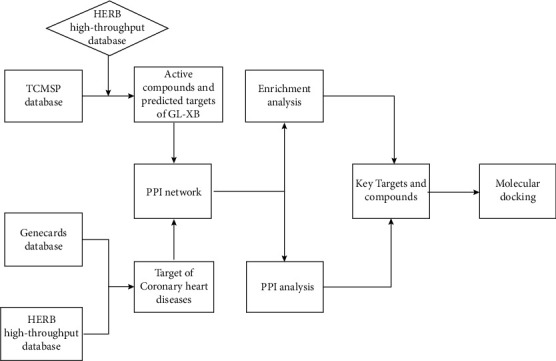
The workflow of our study. TCMSP: Traditional Chinese Medicine Systems Pharmacology Platform; GXLB: Gualou Xiebai decoction; HERB: Bencao Zujian; PPI: psychophysiological interaction.

In 2008, British pharmacologist Andrew Hopkins proposed the idea of network pharmacology, which integrates bioinformatics, multimodal pharmacology, network data analysis, and computer technology to update the current research model from “one target, one drug” to a network-based model. This “drug-compound-target” model offers an improved strategy for exploring associations between various drugs and diseases [4]. Therefore, in the current study, we aimed to analyse the mechanism by which GLXB exerts beneficial effects in the treatment of CHD using a network pharmacology approach. We also performed molecular docking analyses to verify the affinity between the compound and the target.  Figure 1 is the schematic representation of the study workflow.

## 2. Methods

### 2.1. Data Collection and Processing

The Traditional Chinese Medicine Systems Pharmacology Platform (TCMSP) (http://lsp.nwu.edu.cn/) is an open database containing information regarding oral bioavailability (OB), drug similarity (DL), equal-reference values, targets of action, participating pathways, and related diseases for nearly 500 Chinese herbal medicines and their bioactive compounds [5]. The TCMSP was used to screen for GLXB compounds. According to the pharmacokinetic absorption, distribution, metabolism, and excretion parameters, OB ≥ 30%, and DL ≥ 0.18 were the conditions used to screen out the active ingredients.

Bencao Zujian (HERB) (http://herb.ac.cn/) is a high-throughput database established by Professor Zhao Yi and his team [6]. They reanalysed the expression profiles of 6,164 genes in 1,037 high-throughput experiments evaluating herbs/ingredients and manually selected 1,241 gene targets and 494 modern diseases from 1,966 recently published articles. The HERB database is based on objective data and experiments, and relevant experimental support can be found for the interaction between each drug and target, with high reliability. We searched the database using “gualou” and “xiebai” as keywords to obtain the targets of GLXB.

Similarly, we searched the HERB and GeneCards (https://www.genecards.org/) databases using the term “coronary heart diseases” as a keyword to obtain the target of CHD.

### 2.2. Network Construction

We used the graphical software Cytoscape3.6.1 to construct the “compound-target” network diagram for GLXB. In the network diagram, “nodes” represent compounds or action targets, and “edges” represent the association between compounds and targets. First, we obtained the data input software from the TCMSP database to generate the basic network. Next, we examined the intersections of targets in all the databases to obtain the key targets. Finally, we selected the key target in the basic network and the compounds connected to it to obtain the key network.

### 2.3. Enrichment Analysis

Key targets were recorded in the DAVID6.8 database (https://david.ncifcrf.gov/summary.jsp) to obtain the Kyoto Encyclopedia of Genes and Genomes (KEGG) signalling pathway data. Results with *P* values less than 0.05 were considered significant and subjected to further analysis.

### 2.4. Molecular Docking

AutoDock Vina_1.1.2 software was used to conduct molecular docking analyses between major compounds and key targets of potential pathways. AutoDock Vina uses a semiflexible molecular docking model, in which the pharmacophore is flexible while the protein remains rigid during docking. The docking results were evaluated using a semiempirical free energy function.

The specific steps of molecular docking analysis were as follows: first, we downloaded the three-dimensional structure of the active ingredient file (“Spatial Data File” format) from the PubChem website (https://pubchem.ncbi.nlm.nih.gov/). We then downloaded the crystal structure of the key target molecule from the Protein Data Bank website (http://www.rcsb.org/). Subsequently, we used AutoDock tools to separate the target protein and its ligand, added a hydrogen atom, calculated the electric charge, and exported it to the PDBQT format file. Finally, we used Vina to connect the active ingredients with the target protein, and affinity data were extracted. PyMOL was used to analyse and plot the results [7].

## 3. Results

### 3.1. Data Collection

Analysis of the TCMSP database revealed that GL had 11 active compounds and 32 predicted targets, while XB had 21 active compounds and 32 predicted targets. From the HERB database, we identified 78 high-throughput targets for GL and 36 high-throughput targets for XB. We obtained 1,225 and 7,100 CHD targets in the HERB and GeneCards databases, respectively.

### 3.2. Network Diagram

**Figure 2:**
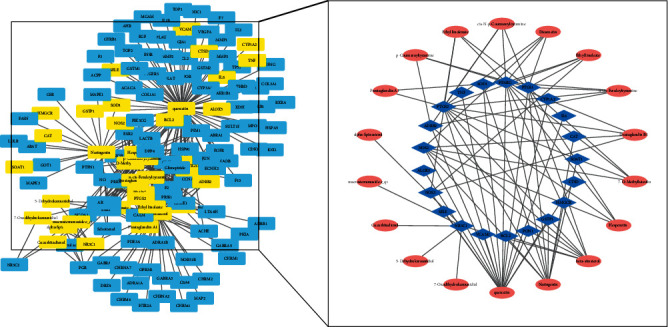
Filtering process for the network diagram. In the figure on the left, the squares represent all compound and protein targets. Among them, yellow squares represent intersection targets and intersection compounds. We extracted the yellow squares and arranged them to obtain the network diagram on the right, in which red ovals and blue diamonds represent the compounds and the targets, respectively.

We input the TCMSP data into the graphical software Cytoscape3.6.1 to generate the basic network. Subsequently, we intersected the targets extracted from all databases, which resulted in 21 key targets. Eighteen key compounds were identified and used to establish a key network by selecting the nodes connected to the key targets. The key network filtering process is illustrated in Figure 2.

### 3.3. Enrichment Analysis

**Figure 3:**
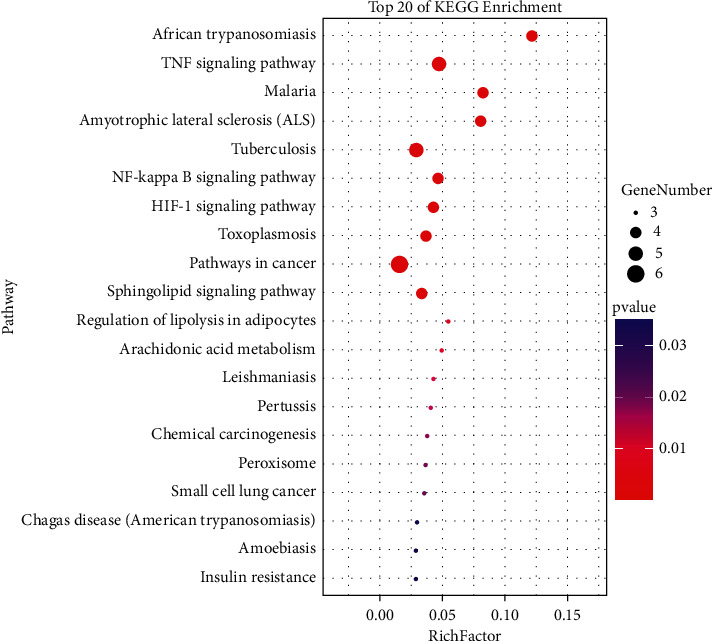
The top 20 results in the enrichment analysis. Among them, the tumour necrosis factor signalling pathway, nuclear factor-kappa B signalling pathway, hypoxia-inducible factor signalling pathway, arachidonic acid metabolism, and insulin resistance were closely associated with coronary heart disease.

KEGG analysis indicated that the key targets of GLXB in the treatment of CHD were enriched in 26 pathways, and 21 of them had *P* values less than 0.05. Among these pathways, the tumour necrosis factor (TNF) signalling pathway, nuclear factor (NF) kappa B signalling pathway, hypoxia-inducible factor (HIF) 1 signalling pathway, arachidonic acid metabolism, and insulin resistance were closely associated with GLXB treatment for CHD. The top 20 pathways identified in the enrichment analysis are shown in Figure 3. We also identified six key targets via reverse targeting in these five pathways: interleukin 6 (1ALU), TNF (2AZ5), prostaglandin-endoperoxide synthase 2 (PTGS2) (5KIR), B-cell leukaemia/lymphoma 2 (BCL2) (4LVT), nitric oxide synthase 3 (3EAH), and vascular cell adhesion molecule-1 (VCAM1) (1VCA).

### 3.4. Molecular Docking Results

**Figure 4:**
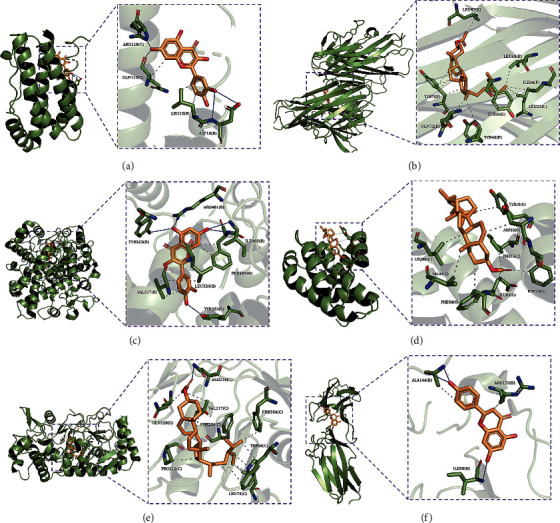
Components with the highest affinity in the molecular docking analysis. (a) Interleukin 6 and quercetin, affinity = −6.3 kcal/mol. (b) Tumour necrosis factor and *β*-sitosterol, affinity = −8.3 kcal/mol. (c) Prostaglandin-endoperoxide synthase 2 and quercetin, affinity = −9.7 kcal/mol. (d) B-cell leukaemia/lymphoma 2 and *β*-sitosterol, affinity = −7.6 kcal/mol. (e) Nitric oxide synthase 3 and *β*-sitosterol, affinity = −9 kcal/mol. (f) Vascular cell adhesion molecule-1 and naringenin, affinity = −6.6 kcal/mol. Solid blue lines represent hydrogen bonds, while dotted grey lines represent hydrophobic interactions.

AutoDock Vina evaluates the binding ability of small molecules to proteins mainly based on affinity. An affinity of less than 0 indicates that the ligand can spontaneously bind to the receptor, and smaller values are indicative of higher binding energy, meaning that the active compound is more easily associated with the receptor. We obtained the compounds associated with the key targets through the network diagram, which included quercetin, naringenin, *β*-sitosterol, ethyl linolenate, ethyl linoleate, and prostaglandin B1. The docking results are shown in Table 1. The compounds with the highest affinity are shown in Figure 4.

## 4. Discussion

In previous centuries, in the absence of modern medical tests, doctors could only diagnose a patient's illness based on his/her appearance. The outward manifestation of CHD is chest discomfort associated with physical activity. TCM works indicate that the herbal medicine GLXB has been used to treat the external manifestations of chest discomfort. In traditional medicine, GLXB is believed to ease the symptoms of chest discomfort by smoothing the meridians. Our study suggests that the beneficial effects of GLXB in patients with CHD may be associated with intervention in pathways related to TNF signalling, NF-kappa B signalling, HIF-1 signalling, arachidonic acid metabolism, and insulin resistance.

Our understanding of CHD has deeply changed in recent years: atherosclerosis is no longer considered a simple lipid storage disorder but is instead regarded as a systemic inflammatory disease. Indeed, inflammation plays a pivotal role in all stages of atherogenesis, including foam cell accumulation, fatty streak organisation, fibrous plaque formation, acute plaque fissuring, rupture, and thrombosis [8].

The NF-kappa B signalling pathway is activated when the organism is stimulated or when reactive oxygen species become unbalanced, thus leading to the release of proinflammatory factors, such as IL-6, IL-8, TNF-*α*, iNOS, ICAM-1, VCAM-1, and cyclooxygenase (COX) 2. At the same time, increased production and release of proinflammatory factors further activate NF-kappa B, which in turn induces NOD-like receptor protein-3 inflammasomes, leading to continuous amplification of initial inflammatory signals and the inflammatory cascade effect [9, 10]. NF-kappa B also plays a central role in the activation of chemotactic cytokines, which contribute to the recruitment of monocytes/macrophages. Research has also indicated that NF-kappa B promotes the transcription and translation of monocyte chemotactic protein-1 and its receptor (chemotactic factor receptor), thereby activating VCAM-1 [11].

VCAM-1 attracts monocytes, which then migrate through the endothelial layer under the influence of various proinflammatory chemoattractants. Once within the arterial intima, the monocytes continue to undergo inflammatory changes, transform into macrophages, engulf lipids, and become foam cells. T lymphocytes also migrate into the intima, where they release proinflammatory cytokines that amplify the inflammatory activity. Inflammatory cells then attach and cross the blood vessel wall into the ischaemic areas of the heart, where they cleave proteases to produce reactive oxygen species and block microvessels, resulting in damage to the heart cells [12].

IL-6 is secreted by T cells, macrophages, and endothelial cells and propagates inflammatory cascades in response to inflammatory stimuli [13]. An animal study has demonstrated that IL-6 administration to apolipoprotein E^–/–^ mice on a high-fat diet can promote plaque formation [14]. In addition, animal studies have found elevated levels of IL-6 in mast cell-deficient mice, suggesting that IL-6 is a mediator of CHD. IL-6 may promote a procoagulant state by upregulating the expression of fibrinogen [15], which is considered a downstream marker of the inflammatory response. In addition to participating in the clotting cascade, fibrinogen is hypothesised to stimulate smooth muscle cell migration, promote platelet aggregation, and increase blood viscosity [16]. Previous studies have shown that fibrin may bind to lipoproteins in blood vessel walls, promoting lipid accumulation in fibrous plaques, and leading to plaque growth [17].

TNF-*α* is a proinflammatory cytokine that can lead to tumour cell necrosis. TNF-*α* is involved in the pathological process of atherosclerosis by increasing inflammatory cells in injured tissues and assisting vascular smooth muscle remodeling [18]. Meanwhile, TNF-*α* can decrease the expression of endothelial NOS, reduce the bioavailability of nitric oxide, and promote oxidative stress by decreasing levels of nitric oxide and endothelial NOS expression. This process intensifies oxidative damage to vascular endothelial cells and promotes plaque formation [19]. TNF-*α* is also an exogenous activator of cell apoptosis [20], and the inhibition of vascular smooth muscle cell death during the development of CHD plays a significant role in reducing the lesion area and promoting cardiac recovery [21].

BCL2 is a key antiapoptotic protein. Studies have shown that apoptotic cells induce an inflammatory response by releasing inflammatory cytokines and an immune response by directly activating dendritic cells. These results suggest that CHD can be treated by overexpressing BCL2 in cardiomyocytes to protect them from apoptosis and inhibit local inflammation [22].

The HIF-1 signalling pathway involves the expression of more than 100 genes that coordinate the adaptive response of tissues to hypoxia. HIF-1, a transcriptional regulator produced in response to hypoxia, is degraded by proteasome action on proline hydroxylase (PHD) under normoxic conditions. However, PHD activity is inhibited under hypoxic conditions; thus, HIF-1 cannot be degraded [23]. HIF-1 plays an important role in angiogenesis, and its downstream genes can induce the production of a variety of angiogenic signalling molecules, including vascular endothelial growth factor, angiotensin (Ang) I, and Ang-II [24]. Therefore, HIF-1 can improve exercise tolerance and quality of life in patients with CHD by promoting the formation of ischaemic myocardial collateral circulation [25]. In addition, studies have shown that HIF-1 can reduce the expression of NF kappa-B and inflammatory factors, thereby reducing the inflammatory response in the myocardium [26].

Arachidonic acid (AA) is the precursor of a series of proinflammatory/proaggregating mediators that are metabolised by COX and lipoxygenases into prostaglandins, thromboalkanes, leukotrienes, and other oxidative derivatives [27]. Among them, prostaglandin E2 and leukotriene B4 can promote the production of IL-6 and increase vascular permeability. Thrombosis A2 affects blood pressure and blood flow and promotes platelet activation and aggregation [28]. COX, which plays an important role in this process, has been identified in three subtypes, but only COX-1 and COX-2 are functional. The former, which is structurally expressed in most normal tissues, is a housekeeping enzyme responsible for a variety of physiological functions and is involved in the formation of thromboxane A2. The latter is rarely expressed in normal tissues but is rapidly induced by cytokines and growth factors [29]. COX-2 is involved in angiogenesis, inhibition of apoptosis, and suppression of the immune response, which are closely associated with CHD. The risk of cardiovascular events associated with COX-2 is correlated with polymorphisms in the gene promoter PTGS2 [30]. Meanwhile, as an antagonist of eicosapentaenoic acid (EPA), AA blocks the regulation of lipid metabolism and the anti-inflammatory and antiplatelet effects of EPA. Evidence suggests that reducing the intravascular level of AA can reduce the risk of adverse cardiovascular events [31].

Resistance of muscle and adipose tissue to insulin regulation leads to elevated plasma insulin and free fatty acid concentrations. This combination stimulates liver very low-density lipoprotein (LDL) triglyceride (TG) secretion, leading to increased plasma TG concentrations and hypertriglyceridemia in insulin-resistant nondiabetic individuals [32]. Elevated levels of IL-6 and TNF-*α* also induce insulin resistance and promote the development of CHD. Accordingly, reducing insulin resistance has proven beneficial in patients with CHD [33].

The current results indicate that the active components of GLXB in the treatment of CHD are mainly quercetin, naringenin, *β*-sitosterol, ethyl linolenate, ethyl linoleate, and prostaglandin B1. Molecular docking results revealed that these components have affinity values less than 0, indicating that they can spontaneously bind to their key targets, thus inhibiting the function of the protein.

Flavonoids have been shown to have significant cardiac benefits, such as inhibiting LDL oxidation and endothelial-dependent vasodilation, reducing levels of adhesion molecules, and other inflammatory markers while protecting nitric oxide and endothelial function under conditions of oxidative stress and preventing oxidation and inflammatory damage to neurons and platelet aggregation [34]. Quercetin and naringenin are flavonoids. Some studies have shown that treatment with 120 mg of quercetin daily can inhibit inflammation and reduce NF-kappa B transcription activity in patients with CHD [35]. Naringenin also inhibits the production of inflammatory factors and NF-kappa B activation [36]. In terms of metabolism, naringenin has been shown to reduce plasma triglyceride and cholesterol levels as well as insulin resistance [37].

The European Food Safety Authority and US Food and Drug Administration recommend that individuals consume 1.5 to 2.4 g of phytosterols daily to lower blood cholesterol levels, as this reduces the risk of heart disease [38]. *β*-Sitosterol is a phytosterol that exerts anti-inflammatory and immunomodulatory effects by reducing plasma IL-6 and TNF-*α* levels [39]. Although few studies have investigated the effects of ethyl linoleate, ethyl linolenic acid, and prostaglandin B1, the current study highlights the potential of these three compounds in the treatment of CHD based on their modes of action and the results of the molecular docking analysis. Further studies are required to determine whether these compounds have clinical significance in the treatment of CHD.

This study had some limitations. Despite the integration of information from the databases screened in our study, we may have missed some compounds that exert positive effects in patients with CHD. At the same time, due to the limitations of network pharmacology, additional studies are required to determine the dose-effect relationships of the compounds identified in the current study.

## 5. Conclusion

Using a network pharmacology approach, the current study identified the material basis and potential mechanisms by which GLXB exerts its effects in the treatment of CHD. This approach conforms to the scenario observed for many TCM formulations, which include multiple compounds that act on multiple targets. Our molecular docking analyses allowed us to determine the affinity between the compounds and their targets, providing preliminary evidence regarding the potential of these compounds in the treatment of CHD. Our study thus provides new insight into screening for active small molecular compounds contained in natural herbs, which is beneficial for the development of new drugs.

## Figures and Tables

**Table 1:** Molecular docking results.

	BCL2	IL-6	NOS3	PTGS2	TNF	VCAM1
Naringenin	−7.2	−6.2	−8.2	−9.4	−7.5	−6.6
Quercetin	−6.9	−6.3	−8.6	−9.7	−7.8	−6.4
*β*-Sitosterol	−7.6	−6.3	−9	−1.4	−8.3	−5.6
Prostaglandin B1	−6.3	−4.7	−7	−7.7	−6.5	−4.2
Ethyl linolenate	−5.4	−4.5	−6.4	−7.3	−5.6	−3.7
Ethyl linoleate	−5.8	−4.7	−6.2	−6.6	−5.4	−3.5

The figures in the table represent the affinity determined in the molecular docking analysis (kcal/mol).

## Data Availability

The data used to support the findings of this study are available from the corresponding author upon request.
